# Impact of Multi-Bias on the Performance of 150 nm GaN HEMT for High-Frequency Applications

**DOI:** 10.3390/mi16080932

**Published:** 2025-08-13

**Authors:** Mohammad Abdul Alim, Christophe Gaquiere

**Affiliations:** 1 Electrical and Electronic Engineering, University of Chittagong, Chittagong 4331, Bangladesh; 2 Institute of Electronic, Microelectronic and Nanotechnology (IEMN), The University of Lille, F-59000 Lille, France; christophe.gaquiere@iemn.univ-lille1.fr

**Keywords:** GaN HEMT, 150 nm gate length, multi-bias characterization, small-signal analysis, on-wafer measurements

## Abstract

This study examines the performance of a GaN HEMT with a 150 nm gate length, fabricated on silicon carbide, across various operational modes, including direct current (DC), radio frequency (RF), and small-signal parameters. The evaluation of DC, RF, and small-signal performance under diverse bias conditions remains a relatively unexplored area of study for this specific technology. The DC characteristics revealed relatively little I_ds_ at zero gate and drain voltages, and the current grew as V_gs_ increased. Essential measurements include I_dss_ at 109 mA and I_dssm_ at 26 mA, while the peak g_m_ was 62 mS. Because transconductance is sensitive to variations in V_gs_ and V_ds_, it shows “Vth _roll-off_,” where Vth decreases as V_ds_ increases. The transfer characteristics corroborated this trend, illustrating the impact of drain-induced barrier lowering (DIBL) on threshold voltage (Vth) values, which spanned from −5.06 V to −5.71 V across varying drain-source voltages (V_ds_). The equivalent-circuit technique revealed substantial non-linear behaviors in capacitances such as C_gs_ and C_gd_ concerning V_gs_ and V_ds_, while also identifying extrinsic factors including parasitic capacitances and resistances. Series resistances (R_gs_ and R_gd_) decreased as V_gs_ increased, thereby enhancing device conductivity. As V_gs_ approached neutrality, particularly at elevated V_ds_ levels, the intrinsic transconductance (g_mo_) and time constants (τ_gm_, τ_gs_, and τ_gd_) exhibited enhanced performance. f_t_ and f_max_, which are essential for high-frequency applications, rose with decreasing V_gs_ and increasing V_ds_. When V_gs_ approached −3 V, the S_21_ and Y_21_ readings demonstrated improved signal transmission, with peak S_21_ values of approximately 11.2 dB. The stability factor (K), which increased with V_ds_, highlighted the device’s operational limits. The robust correlation between simulation and experimental data validated the equivalent-circuit model, which is essential for enhancing design and creating RF circuits. Further examination of bias conditions would enhance understanding of the device’s performance.

## 1. Introduction

The beneficial properties of GaN HEMTs have made them a focal point in the semiconductor industry. Due to their high breakdown voltage, high electron mobility, and high thermal conductivity, they are suitable for high-power and high-frequency applications [1,2,3,4]. Compared to conventional silicon and GaAs devices, GaN HEMTs that utilize silicon carbide (SiC) substrates offer even greater benefits. SiC’s exceptional thermal conductivity enhances the efficiency and power density of various systems, including radar systems and RF amplifiers, by effectively dissipating heat during high-power operation [5,6,7,8,9,10]. Despite numerous studies examining the DC and RF performance characteristics of GaN HEMTs [11,12,13,14,15,16,17,18,19,20,21,22,23,24], there is limited information available regarding their behavior under varying bias conditions, especially concerning small-signal and multi-bias performance [25,26,27,28,29,30]. To design and analyze GaN RF circuits, it is essential to investigate how multi-bias conditions influence crucial parameters, including transconductance (g_m_), output resistance (R_ds_), and intrinsic capacitances. Existing literature has primarily focused on basic DC characteristics under static bias conditions, with earlier work addressing only the influence of bias on switching ability and efficiency. Prior research has focused on the effects of passive and active parasitics, leaving the dynamic impact of bias on RF and small-signal parameters less studied. For example, some research has examined the effects of parasitic components on frequency response and stability [31,32,33], while other research has focused on bias-related noise performance [14,34,35]. However, there has not been a thorough examination of DC, RF, and small-signal characteristics under various bias conditions. The objective of this research is to investigate the behavior of a GaN HEMT 150 with a 150 nm gate length, built on a SiC substrate, and to highlight the impact of bias conditions on equivalent circuit components and high-frequency performance.

Key DC results that highlight the device’s performance capabilities include I_dss_, which was measured at 109 mA, and I_dssm_, which was measured at 26 mA. The device’s sensitivity to these biasing parameters is further illustrated by the transconductance (g_m_), which attained 62 mS at V_gs_ = −4.8 and V_ds_ = 10 V. Due to short-channel effects, such as DIBL, the threshold voltage (Vth) decreased as V_ds_ increased, with values ranging from −5.06 V to −5.71 V. At higher V_ds_, the dark current (I_dso_) increased exponentially, indicating an increase in carrier generation. An equivalent circuit analysis shows how extrinsic elements, such as parasitic resistances and capacitances, affect performance. Biasing ranged from V_gs_ = −6.0 V to −3.0 V and from Vds = 3 V to 15 V, with a focus on frequencies between 45 MHz and 50 GHz. The extraction process for device parameters starts by identifying extrinsic components to determine intrinsic parameters. C_gs_ increased with both V_ds_ and V_gs_, while C_gd_ decreased. Simultaneously, C_ds_ rose as V_gs_ neared pinch-off. Owing to non-quasi-static phenomena, the series resistances R_gs_ and R_gd_ exhibited charging delays, whereas R_ds_ increased with V_ds_, necessitating a balance for optimal design. At V_ds_ = 11 V and V_gs_ = −4.8 V, the intrinsic transconductance g_mo_ reached a maximum of 71 mS. It escalates with V_gs_. Transistor inertia at elevated frequencies is indicated by intrinsic g_mo_ and time constants (τ_gm_, τ_gs_, and τ_gd_), with g_mo_ reaching its maximum at reduced V_gs_ as V_ds_ increased. Increased V_ds_ and decreased negative V_gs_ yielded enhanced frequency performance metrics, with f_T_ ranging from 1.18 × 10^8^ Hz to 5.16 × 10^10^ Hz and f_max_ from 5.23 × 10^8^ Hz to 9.96 × 10^10^ Hz. The S_21_ parameter peaked at 11.2 dB with V_gs_ = −4.8 V and V_ds_ between 11 and 15 V. Furthermore, the stability factor K increased with higher V_ds_ and lower V_g_, reaching a maximum of approximately 1.3, indicating unconditional stability. This work utilises small-signal parameters derived from measured S-parameters in ADS software 2022 to compare simulations with measurements through an equivalent circuit model.

This research aims to investigate how biasing influences GaN HEMTs, particularly their small-signal operation and RF performance. It proposes a systematic method for evaluating circuit components under varying bias conditions, which is crucial for optimizing GaN HEMTs for high-power and high-frequency applications, thereby advancing semiconductor technology.

## 2. Fabrication and Measurements

**Figure 1:**
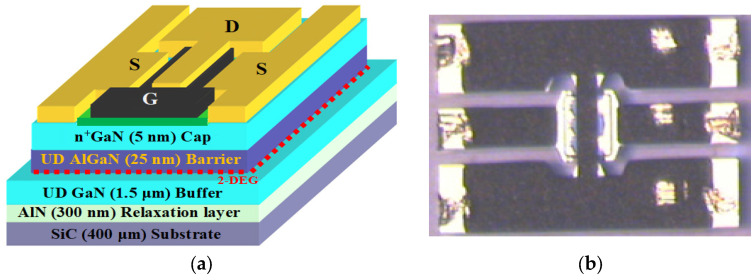
(**a**) Cross-section view of AlGaN/GaN/SiC HEMT structure (not to scale); (**b**) optical micrographs [36].

This study aims to investigate a HEMT based on an AlGaN/GaN heterostructure [36] grown by metalorganic vapor-phase epitaxy (MOVPE) on a 400-µm-thick silicon carbide substrate, as in Figure 1. A GaN buffer layer was incorporated into the design to achieve a 2-DEG at the AlGaN-GaN junction, which relies on the charge induced by polarization between the AlGaN and GaN layers, thereby providing a good number of electrons without the need for impurity doping. The device utilized a 20 nm Al0.253Ga0.747N barrier and a 1.5 µm GaN buffer epitaxial layer to achieve a good lattice match with the substrate (SiC). This was succeeded by a GaN channel layer and a very thin 5 nm AlGaN spacer layer, which also enables high-speed electron motion and facilitates the modulation of charge density through control of the gate bias voltage applied. To obtain low-resistance ohmic contact at the source and drain regions, a thin 5 nm cap layer of GaN was deposited. Important parameters recorded include the sheet resistance (RSH) of 324 Ω/□ from Hall measurements, and TLM showed the contact resistance (Rt) to be 0.36 Ω·mm. In Hall measurements, the charge density (N_S_) was greater than 1.3 × 10^13^ cm^−2^, and the most significant electron mobility was higher than 1400 cm^2^/V·s. The gate configuration comprised four 50 µm fingers, resulting in an overall gate width of 200 µm and a gate length of 0.15 µm. Ohmic contacts of Ti/Al/Ni/Au were created by thermally annealing at 900 °C. The mushroom-shaped Schottky gate consisted of a Pt/Ti/Pt/Au stack, which helps reduce the gate capacitance and consequently enhances the control of transconductance, while decreasing leakage and noise. The device was encapsulated with a 240 nm thick Si_3_N_4_ layer, deposited by PECVD at 340 °C, to further enhance efficiency and reliability. The transistors were manufactured using the UMS GH15 process. They were created through Molecular Beam Epitaxy (MBE) at IEMN in the University of Lille, France.

**Figure 2:**
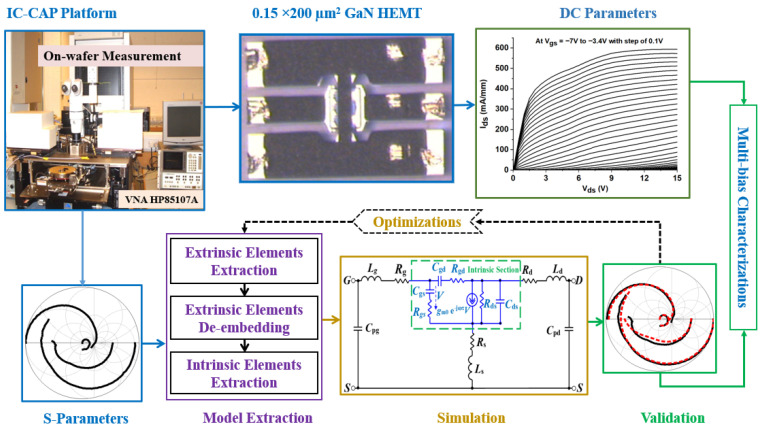
Diagram depicting the methodology for quantifying and assessing 150 nm GaN HEMT.

Four main steps comprise the device characterization process as illustrated in Figure 2: (1) S-parameter acquisition, (2) cold pinch-off technique, (3) de-embedding, and (4) ADS simulation flow. First, an HP8510C Vector Network Analyzer (VNA), with the aid of an HP4142B DC source, supplied the required current, connected via Ground-Signal-Ground (GSG) radio frequency probes that spanned 45 MHz to 50 GHz, to measure S-parameters. Multiple biasing conditions (V_gs_ from −6 V to –3 V and V_ds_ from 3 V to 15 V) were used for the measurements, and IC-CAP software 2022 was employed to automate data acquisition and ensure accuracy. In the second step, extrinsic parasitic components like inductances (L_g_, L_d_, L_s_), resistances (R_g_, R_s_, R_d_), and pad capacitances (C_pg_, C_pd_) could be accurately extracted by suppressing intrinsic device activity using the cold pinch-off technique with V_gs_ = −10 V and V_ds_ = 0 V. Third, by gradually modifying and eliminating capacitance values (C_pg_ and C_pd_), a de-embedding procedure was carried out to eradicate parasitic effects, especially the PDRZ effect, until the frequency-dependent anomalies in Re (Zij) were removed. Inductive and resistive parameters were also extracted using OFF-state bias data (V_gs_ = 0 V, V_ds_ = 0 V). A comprehensive small-signal equivalent circuit model was then constructed by importing the cleaned and bias-dependent S-parameter data into the Advanced Design System (ADS). Both extrinsic and intrinsic components (g_m_, C_gs_, C_gd_, C_ds_, R_gs_, R_gd_, and R_ds_) were included in this model, which was validated through simulations under all tested frequencies and bias conditions.

## 3. Results and Analysis

The operation of HEMTs can be described across different voltage regions. In the sub-threshold region, when V_gs_ is less than V_th_, the current increases exponentially with V_gs_, but is small enough, making it suitable for ultra-low-power device applications. When the dependence of V_gs_ surpasses Vth and V_ds_ is adequately elevated, the square law region fluctuates, yielding a current that is roughly equivalent to V_gs_ squared. This region is optimal for amplifiers due to the advantageous correlation between V_gs_ and current, making it a preferred choice in analog circuit designs. In this instance, the transconductance (g_m_) increases with current. In contrast, elevated V_gs_ values lead to the saturation of carrier velocity within the velocity saturation region, maintaining constant transconductance and diminishing the increase in current, thereby affecting performance. When selecting V_gs_ = −3.0 V to −7.0 V and V_ds_ = 3 V to 15 V, it is recommended that V_gs_ be maintained above the threshold level for maximum amplification, and V_gs_ should not enter the velocity saturation region. Regarding the square-law region, its emphasis enables most of the HEMT’s characteristics to be effectively exploited, which is why it is suitable for use in high-performance analog circuits.

### 3.1. DC Behavior with Biasing

**Figure 3:**
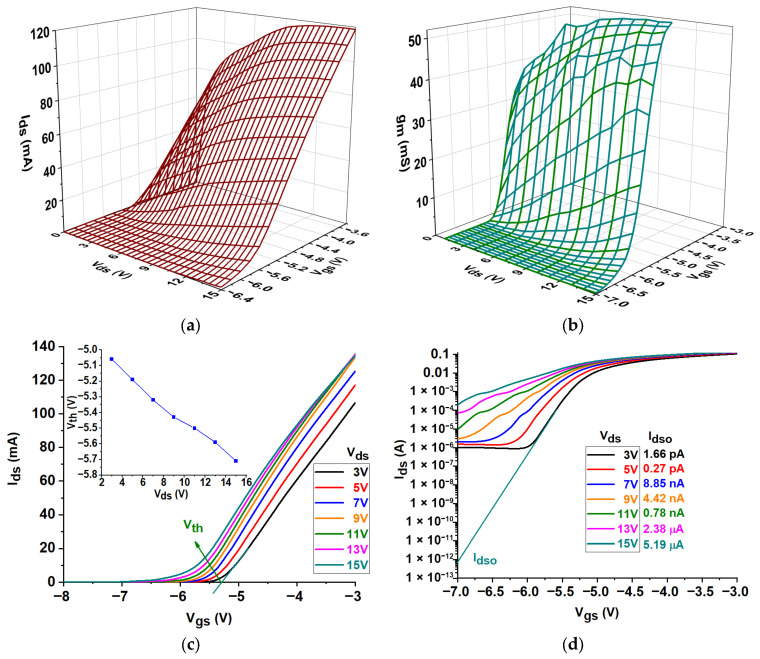
(**a**) DC characteristics, (**b**) transconductance characteristics, (**c**) transfer characteristics, and (**d**) semi-logarithmic transfer characteristics.

Figure 3a illustrates the DC attributes of a GaN HEMT. Even with V_ds_ and V_gs_ equal to zero, there is practically no drain current (I_ds_), which is usually less than the reverse leakage current [16]. As the gate voltage increases, the current through the drain also increases. The data can be divided into two main regions: the triode region, characterized by a linear increase in I_ds_ with rising V_ds_, and the saturation region, where Ids becomes limited or saturated once V_ds_ reaches a specific threshold due to channel constriction at the drain side [37]. From the I–V graph, two key metrics emerge: I_dss_, the drain current when the transistor was saturated at V_gs_ = −3.0 V, measured at 109 mA, and I_dssm,_ the maximum drain-source current at peak transconductance, recorded at 26 mA. Figure 3b,c show the transconductance (g_m_) and transfer characteristics (g_m_ versus V_gs_ and I_ds_ versus V_gs_). The maximum transconductance achieved was 62 mS at V_gs_ = −4.8 V and V_ds_ = 10 V, resulting in a ratio of 4.23 between I_dss_ and I_dssm_, which indicates optimal small-signal gain [38]. The maximum transconductance values were determined at different combinations of V_gs_ and V_ds_. The measurements revealed a transconductance of 46.23 mS with V_gs_ at −4.6 V and V_ds_ at 3 V. A transconductance of 54.48 mS was observed at a V_gs_ of −4.6 V and a V_ds_ of 6 V. The peak transconductance of 59.62 mS was attained at a V_gs_ of −4.7 V and a V_ds_ of 9 V. A value of 57 mS was recorded at V_gs_ of −4.8 V and V_ds_ of 12 V. A transconductance of 56.8 mS was observed at a V_gs_ of −4.9 V and a V_ds_ of 15 V. These values underscore the sensitivity of transconductance to variations in both V_gs_ and V_ds_ within the designated ranges. As V_ds_ increased from 3 V to 15 V, the peak transconductance (g_m_) changed from −4.6 V to −4.9 V. This is because threshold voltage (Vth) varies with V_ds_, a phenomenon known as “Vth roll-off” [39]. The roll-off has a significant impact on how well the device performs. As V_ds_ increases, Vth decreases, which means that V_gs_ must be adjusted to maintain the desired conduction levels. This behavior improves transconductance and overall efficiency between −4.6 V and −4.9 V. These differences demonstrate the importance of carefully managing circuit design to ensure that biasing, gain, and overall reliability are stable. Figure 3c shows that the threshold voltage (Vth) values had a consistent trend in the transfer characteristics (I_ds_ vs. V_gs_). The Vth values were −5.06 V at V_ds_ = 3 V, −5.19 V at V_ds_ = 5 V, −5.32 V at V_ds_ = 7 V, −5.43 V at V_ds_ = 9 V, −5.50 V at V_ds_ = 11 V, −5.59 V at V_ds_ = 13 V, and −5.71 V at V_ds_ = 15 V. The decrease in Vth as V_ds_ rises suggests that higher drain-source voltages lower the threshold voltage, possibly because of short-channel effects like drain-induced barrier lowering (DIBL) [40]. This observation is essential for understanding device performance in low-voltage applications. V_ds_ and effective channel length influence Vth in short-channel HEMTs but remain unaffected by channel width [41]. The semi-log transfer curve in Figure 3d shows the drain-to-source dark current (I_dso_) measurements, indicating a notable variation in current levels as V_ds_ increased. The dark currents observed were 1.66 pA, 0.27 pA, 8.85 nA, 4.42 nA, 0.78 nA, 2.38 µA, and 5.19 µA for V_ds_ values of 3 V, 5 V, 7 V, 9 V, 11 V, 13 V, and 15 V, respectively. This pattern suggests an exponential growth in dark current with increasing V_ds_, possibly indicating enhanced carrier generation or reduced barrier potential at higher voltages [42,43].

### 3.2. Equivalent Circuit Parameters with Biasing

**Figure 4:**
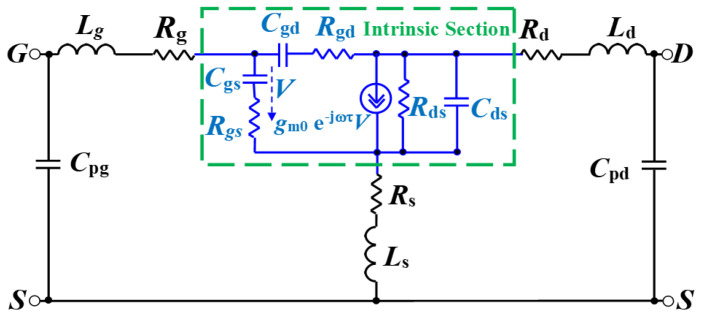
The equivalent circuit model for the investigated GaN HEMT.

**Figure 5:**
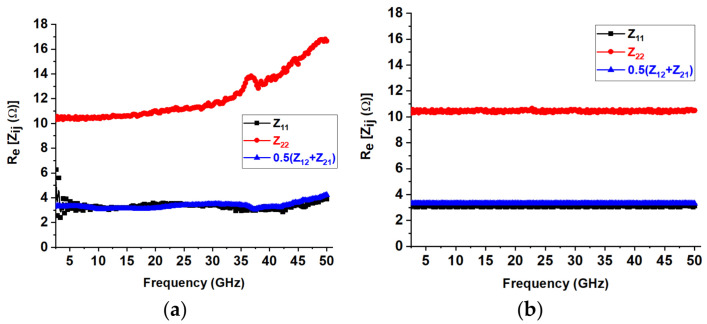
Re (Z_ij_) against frequency under zero bias condition: (**a**) before and (**b**) after de-embedding of the extrinsic capacitances.

The equivalent-circuit approach [44], outlined in Figure 4, is employed to evaluate the S-parameters of the device. The equivalent-circuit parameters (ECPs) were determined using the “cold” pinch-off technique, a method that has been successfully used in GaN technology for many years. Our analysis focused on the extrinsic parameters of the semiconductor device, particularly the parasitic capacitances (C_pg_ and C_pd_), parasitic inductances (L_g_, L_d_, and L_s_), and terminal resistances (R_s_, R_g_, and R_d_), among other factors. When the device was under pinch-off bias conditions (V_ds_ = 0, V_gs_ = −10 V), it was observed that the parasitic capacitances primarily affected the imaginary part of the Y parameters. We also examined the frequency dependence of these capacitances. Figure 5 depicts the behavior of Re (Zij) for the GaN device as a function of frequency under “unbiased” conditions. The device exhibited the PDRZ effect [45], with Re (Zij) starting to rise significantly for the GaN around 36 GHz. The values of Re (Zij) continued to rise with increasing frequency after this onset frequency. The PDRZ effect could be removed from the de-embedded data by deducting the appropriate values of C_pg_ and C_pd_ from the measured data and gradually increasing them from zero until the effect was eliminated. To extract the extrinsic inductances, we used hypothetical Z parameters at OFF bias conditions (V_ds_ = 0, V_gs_ = 0 V) across a specific frequency range. The results showed that inductance L_g_ was significantly more prominent in devices with longer gate lengths. Additionally, it was found that the terminal resistances were heightened due to the influence of the contact metal in the semiconductor. Amongst the three terminal resistances, R_g_ was the smallest, reflecting the effects of metallization in this device. Table 1 presents all the extrinsic parameters of the device.

**Figure 6:**
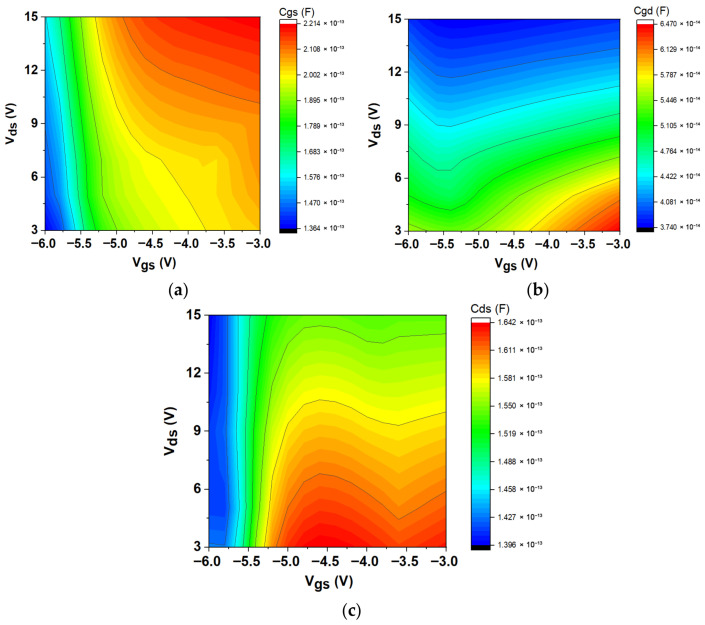
Variation in intrinsic capacitances plotted at different *V*_ds_ and V_gs_ for the AlGaN/GaN/SiC HEMT: (**a**) C_gs_, (**b**) C_gd_, and (**c**) C_ds_.

**Figure 7:**
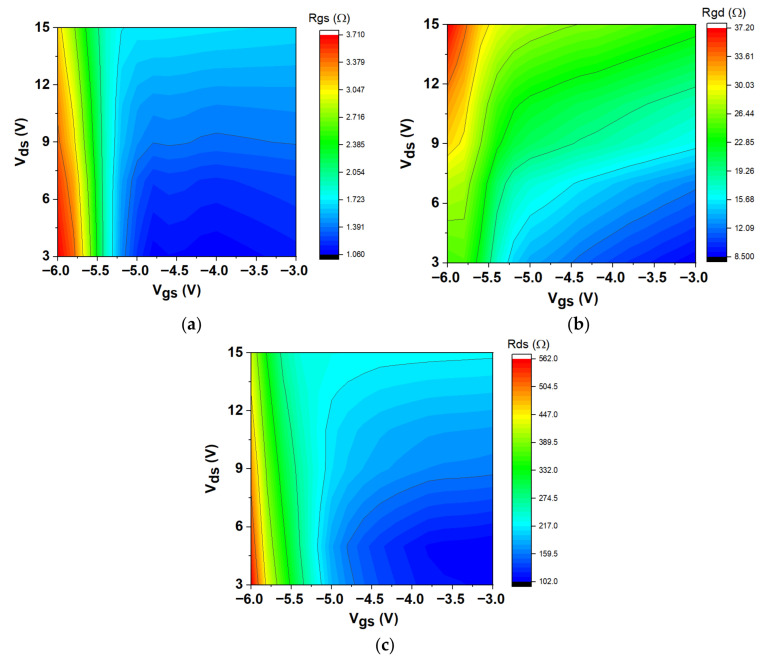
Variation in intrinsic resistances plotted at different *V*_ds_ and V_gs_: (**a**) R_gs_, (**b**) R_gd_, and (**c**) R_ds_.

The initial phase of the extraction process is dedicated to identifying the extrinsic parameters. The intrinsic parameters can be established once these parameters are minimized. The applied bias ranged from V_gs_ = −6.0 V to −3.0 V and V_ds_ = 3 V to 15 V, with intrinsic parameters being extracted between 45 MHz and 50 GHz. Figure 6a shows that the gate-source capacitance, C_gs_, went up when both V_ds_ and V_gs_ went up. C_gs_ stands for the capacitance between the gate electrode and the channel. It shows very non-linear behaviour. It includes both depletion and inversion charges, which are essential for determining the extrinsic cut-off frequency. C_gs_ decreases as V_gs_ decreases owing to the widening of the gate-source depletion region at higher negative voltages [46]. Furthermore, C_gd_ represents the feedback capacitance between the gate and drain, as shown in Figure 6b. Remarkably, this capacitance decreased at higher V_ds_, contrasting with the lower C_gd_ values observed at high negative V_gs_ across varying V_ds_. As V_gs_ increased at a constant V_ds_, C_gd_ gradually rose. The subsequent capacitance analyzed is the drain-source capacitance, C_ds_, depicted in Figure 6c with V_gs_ and V_ds_. As V_gs_ rose from −6 V to −3 V, the C_ds_ values demonstrated a significant increase, especially at lower V_ds_ values. The capacitance substantially rose as V_gs_ transitioned from −6 V to −5.2 V, indicating a substantial effect of gate voltage on capacitance. The effect of changing V_ds_ is clear, especially in the middle range of V_gs_. On the other hand, changing C_ds_ values has a bigger effect when V_gs_ changes. The series resistances, R_gs_ and R_gd_, are derived from the real components of Y_11_ and Y_12_, respectively. The charging delay resulting from non-quasi-static effects is depicted by the distributed channel resistances R_gs_ and R_gd_ [47] in Figure 7a,b. As V_gs_ went from −6 V to −3 V, the R_gs_ resistance values showed a clear downward trend. The resistance was relatively high when V_gs_ was low, such as around −6 V. However, it dropped significantly as V_gs_ increased, especially between −5.4 V and −4.8 V. This behavior indicates that within this range, elevated gate voltages result in lower resistance. The overall trend suggests a more pronounced decline with increasing V_gs_, even though variations in V_ds_ also affect the resistance values. As V_gs_ rose from −6 V to −3 V, the R_gd_ values typically exhibited a declining trend. At reduced V_gs_ values, especially at −6 V, the resistance was comparatively elevated, reaching its maximum at increased V_ds_ levels. As V_gs_ rose, particularly between −5.4 V and −4.8 V, R_gd_ declined considerably, indicating that elevated gate voltages result in reduced resistance. Although changes in V_ds_ affected R_gs_, the main trend indicates that V_gs_ had the most significant impact on resistance characteristics. The “anomalous dip” in S-parameters is more pronounced in devices connected in series to gate-drain capacitance with higher effective gate-drain channel resistance (R_gd_).

As the V_gs_ rose from −6 V to −3 V, the R_ds_ values usually showed a decreasing trend, as shown in Figure 7c. R_ds_ was relatively high at lower V_gs_ values (roughly −6 V), indicating that the device was less conductive. R_ds_ dramatically decreased as V_gs_ increased, particularly between −5.4 V and −4.8 V, suggesting that the device has better conductivity at higher gate voltages. The overall pattern indicates that higher V_gs_ values result in lower R_ds_, underscoring the influence of gate control on the device’s conductivity. In addition, the R_ds_ values vary with V_ds_.

**Figure 8:**
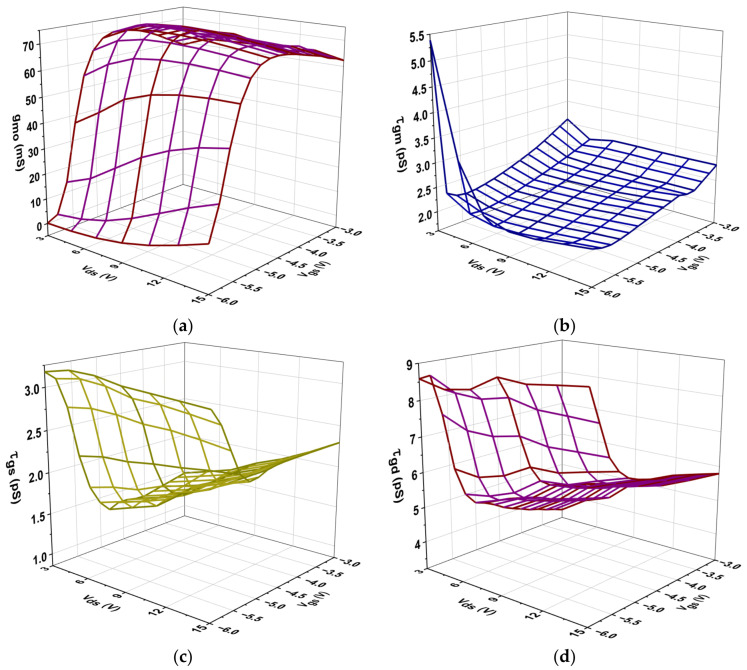
Variation in intrinsic parameters plotted at different V_ds_ and V_gs_ for the AlGaN/GaN/SiC HEMT: (**a**) g_mo_, (**b**) τ_gmo_, (**c**) τ_gs_, and (**d**) τ_gd_.

Figure 8 illustrates the intrinsic transconductance (g_mo_) alongside three intrinsic time constants (τ_gm_, τ_gs_, and τ_gd_). The time constants arise from the transistor’s inherent delay in reacting to rapid signal variations and represent the intrinsic non-quasi-static (NQS) effects that become increasingly pronounced at elevated frequencies. As V_gs_ transitioned from −6 V to −3 V, g_mo_ typically rose across all V_ds_ levels, signifying improved conductivity and the creation of channels within the transistor. The g_mo_ values were noticeably low at V_gs_ = −6 V, suggesting that there was insufficient gate voltage for strong channel formation. On the other hand, g_mo_ attained significantly greater values at V_gs_ = −3 V, peaking at roughly 0.071 mS at V_ds_ = 11 V, suggesting adequate amplification. The data indicate that as V_ds_ increases, g_mo_ tends to stabilize, signifying that the transistor entered the saturation region where further increases in V_ds_ have a decreasing impact on g_mo_. The time delay τ_gm_ in a transistor circuit indicates that as V_gs_ becomes less negative, τgm typically decreases. This means that charge carriers can move around more easily and respond faster. The time delay increased when Vgs was lower, such as when it was close to −6 V, because the conductivity was insufficient. On the other hand, values close to −3 V showed minimal delay, which is perfect for quick switching.

Furthermore, increasing the V_ds_ can improve performance, although delays may stabilize at elevated levels due to saturation effects. This relationship is crucial for designing high-speed circuits. The intrinsic time delay values, τ_gs_, ranged from approximately 3.18 × 10^−12^ s at V_gs_ = −6 V and V_ds_ = 3 V to about 1.29 × 10^−12^ s as V_gs_ approached −3 V with increasing V_ds_. This pattern implies that lower τ_gs_ is linked to higher V_gs_, suggesting a quicker transistor response, particularly at higher V_ds_ levels. The intrinsic time delay values τ_gd_ ranged from approximately 8.59 × 10^−12^ s at V_gs_= − 6 V and V_ds_ = 3 V to about 3.47 × 10^−12^ s at V_gs_ = −3 V and V_ds_ = 3 V. A quicker gate-drain response was indicated by decreasing τ_gd_ values as the V_gs_ became less negative. According to this pattern, improved transistor switching speeds—which are essential for high-frequency applications—are a result of higher V_gs_.

**Figure 9:**
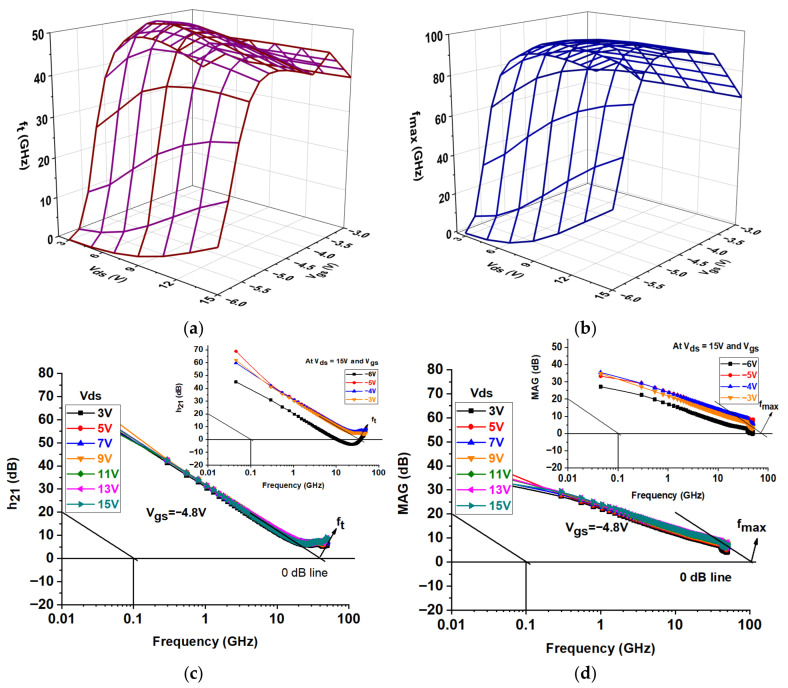
Variation in cut-off and maximum frequencies: (**a**) f_t_, (**b**) f_max_, (**c**) h_21_, (**d**) MAG.

Even in the presence of parasitic effects, Figure 9 demonstrates that the measured h_21_ and MAG exhibited ideal behaviour, declining with frequency at a rate of −20 dB/decade. The f_t_ and f_max_ obtained from the data are displayed in the inset images in Figure 9c,d, which show an inversion at various V_ds_ values. When V_ds_ was higher and V_gs_ was less negative, the cutoff frequency (Figure 9a) typically increased, indicating improved device performance. The range of values was approximately 1.18 × 10^8^ Hz to 5.16 × 10^10^ Hz. Better device performance under these bias conditions was indicated by the f_max_ (Figure 9b), which normally increases with increasing V_ds_ and decreasing negative V_gs_. The range of the f_max_ values was approximately 5.23 × 10^8^ Hz to 9.96 × 10^10^ Hz. The transistor’s suitability for high-frequency applications, such as RF amplifiers and high-speed switching, is demonstrated by both f_t_ and f_max_ [48].

**Figure 10:**
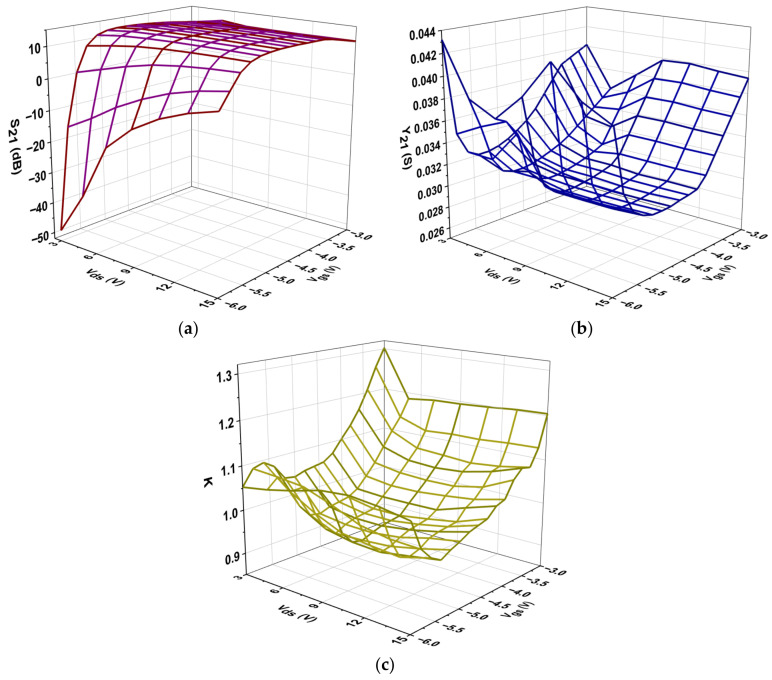
Variation in the intrinsic parameters: (**a**) S_21_, (**b**) Y_21_, and (**c**) K.

In Figure 10, the impact of biasing on the real part of Y_21_, the magnitude of S_21_, and the stability factor K, is illustrated. It is essential to note that the low-frequency values of both Y_21_ and S_21_ are specifically correlated with the intrinsic transconductance g_mo_, as depicted below [49]:

(1) $$Y_{21}=\frac{g_{mo}}{1+g_{mo}R_{s}+g_{DS}\left(R_{S}+R_{D}\right)}$$

(2) $$S_{21}=-2Y_{21}\left(Z_{0}//Y_{22}^{-1}\right)$$

where Z_0_ equals 50 ohms, which is generally much smaller than 1/Y_22_.

The S_21_ parameter indicates that the values were highly negative at lower V_gs,_ such as −6 V, but increased significantly as V_gs_ approached −3.0 V, reflecting improved signal transmission. The highest S_21_ values, approximately 11.2 dB, occurred when V_gs_ = −3.2 V and V_ds_ ranged from 11 to 15 V. Additionally, beyond a certain point, higher V_ds_ values resulted in smaller gains, indicating the transistor’s limitations. As the drain-source voltage rose, the Y_21_ values typically increased with higher V_ds_ across the various V_gs_ values, indicating improved signal transmission. The Y_21_ values tended to rise as V_gs_ became less negative (going from −6 V to −3 V), suggesting improved conductivity and transmission properties of the transistor. The values, which fell between roughly 0.032 and 0.043, indicate that the transistor exhibits good signal amplification capabilities within the tested voltage range. As Vds rose, the stability factor K usually increased as well. This was especially noticeable at V_gs_ = −3 V, where K peaked at about 1.30. Conversely, lower V_gs_ values exhibited a decreasing trend in K for most V_ds_ levels, particularly at V_gs_ = −6 V, where K initially started at around 1.05 but increased slightly at higher V_ds_ values.

**Figure 11:**
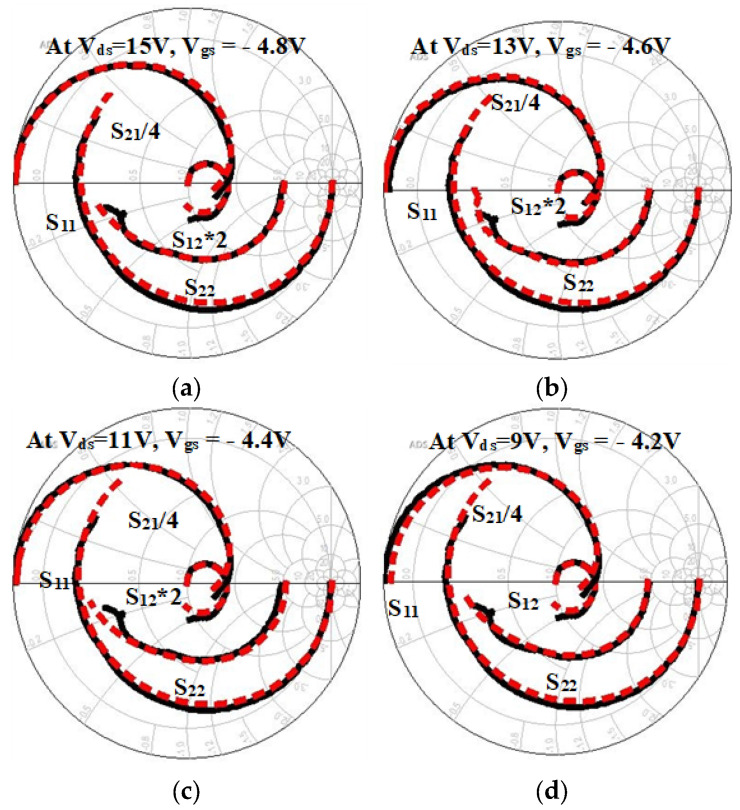
Verification of experimental (black) and modeled (red) data: (**a**) at V_ds_ = 15 V, V_gs_ = −4.8 V, (**b**) at V_ds_ = 13 V, V_gs_ = −4.6 V, (**c**) at V_ds_ = 11 V, V_gs_ = −4.4 V, and (**d**) at V_ds_ = 9 V, V_gs_ = −4.2 V.

The equivalent-circuit model’s precision in depicting the device’s performance under these conditions is corroborated by the robust correlation between simulations and experimental data (Figure 11). Table 2 shows the coefficient of determination (R^2^) and root mean square error (RMSE) values for each bias setting. This was to verify that the simulated and experimental results are in agreement. The model’s accuracy was validated by the low RMSE and high R^2^ values; the former indicates the magnitude of error between the simulated and observed S_21_ values, while the latter demonstrates the robustness of the linear correlation. This validation is essential for assessing practical performance, optimizing design, and advancing complex RF integrated circuit designs.

The data in Table 3 illustrate the performance of the GaN HEMT at varying V_ds_ with a constant V_gs_ of −4.8 V. The I_ds_ consistently rose from 21.81 mA to 54.71 mA as V_ds_ increased from 3 V to 15 V, suggesting better conduction and increased amplification potential. The transconductance (g_m_) peaked at 61.25 mS at 7 V, and the intrinsic g_mo_ also peaked at 73.77 mS under the same conditions. This indicates that the output current control is functioning properly. The C_gd_ went down, which is good because it would reduce the Miller effect and make things more stable. However, the C_gs_ went up, which could mean that switching speeds would be slower. The resistances of R_gs_ and R_gd_ also increased, which could alter the input impedance and the amount of power used. Higher distributed resistance and a delayed charge response are likely causes of the increasing trend of R_gd_ with V_ds_, resulting from non-quasi-static effects and increased voltage stress across the gate-drain region at higher drain biases.

R_ds_ showed some fluctuation, which could affect efficiency and heat generation. Time constants (τ_gm_, τ_gs_, τ_gd_) also increased with V_ds_, resulting in longer response times and potentially limiting high-speed performance. f_t_ reached its highest point at 51.62 GHz at 7 V, and f_max_ rose to 98.90 GHz at 11 V. This means that the device can work at high frequencies. Overall, the forward gearbox gain, S_21_, increased slightly, indicating that amplification remained stable. The forward transconductance parameter, Y_21_, remained constant, indicating that the device remained stable. The stability factor K remained close to 1, indicating that the entire device operated effectively.

## 4. Discussions and Future Directions

This study thoroughly investigates the performance of the 150 nm AlGaN/GaN HEMT on a SiC substrate in the DC, RF, and small-signal regimes. Essential conclusions are emphasised, including the necessity for precise V_ds_ control in high-frequency applications and the threshold voltage’s (Vth) susceptibility to the drain-source voltage (V_ds_), which signifies short-channel phenomena such as DIBL. The study found that transconductance (g_m_ and g_mo_) also changed with bias points, which suggests that charge transport is not linear. The highest intrinsic transconductance occurred at V_gs_ = −4.8 V and V_ds_ = 11 V, which was when the small-signal gain was at its highest. This highlights the importance of parasitic elements, such as capacitances (C_pg_, C_pd_) and resistances (R_g_, R_s_, R_d_), and illustrates how they affect high-frequency performance and frequency-dependent behaviour. Capacitance trends also indicate that increasing V_gs_ and V_ds_ could enhance forward gain and mitigate the Miller effect. This can improve high-frequency parameters, such as f_t_ and f_max_. The research illustrates the influence of intrinsic resistances on conductivity and switching times, indicating that enhancements in resistance at reduced negative gate biases facilitated expedited carrier movement and diminished RC time delays. The S-parameter and Y-parameter responses confirm that signal transmission was improved and the RF amplifier performed well in various situations. Ultimately, the study offers essential insights for future RF circuit designs by validating the equivalent circuit model through substantial concordance with both measured and simulated results.

Future research must focus on several critical domains. To determine how GaN HEMTs will perform over time, they must undergo accelerated ageing tests, particularly under various biasing conditions. This will help us understand how the devices break down, how they fail, and how stable their performance is in high-power, high-frequency applications. A comparative analysis of GaN HEMT performance on various substrates, including SiC, Sapphire, and Si, could also shed light on the trade-offs between power handling, efficiency, cost, and scalability. Additionally, because non-linearities and parasitic effects become more pronounced at higher frequencies, future research should focus on enhancing thermal management, developing innovative packaging technologies, and optimizing device design to ensure that devices operate effectively in the mmWave and terahertz ranges. We also need better methods to address issues with device uniformity that arise due to fabrication tolerances. These tolerances are necessary to ensure that devices function consistently across a wide range of commercial applications. Ultimately, future research should incorporate noise figure measurements and power handling tests to gain a deeper understanding of how GaN HEMTs function. To further enhance the dependability and power handling capabilities of GaN HEMTs for high-frequency applications, effective thermal management techniques should be employed.

## 5. Conclusions

The detailed analysis reveals that the GaN HEMT device has considerable potential for high-frequency applications. It also illustrates the complexity of the relationship between performance metrics and biasing conditions. When there was no bias, the device had a low drain current. As the gate voltage (V_gs_) increased, the drain current (I_ds_) also increased. There were two distinct operating regions: the triode and saturation regions. Key metrics, such as Idss and maximum transconductance (g_m_), show that the device could significantly amplify signals, especially when the bias levels are just right. The observed decrease in threshold voltage (Vth) as the drain-source voltage (V_ds_) increased indicates that drain-induced barrier lowering (DIBL) and other important short-channel effects were occurring. To ensure the device works properly, these effects must be kept in check. Additionally, the dark current’s exponential growth with higher V_ds_ suggests that more carriers were being generated, which could render high-voltage applications less reliable. Finding the equivalent circuit parameters reveals how parasitic components impact a device’s operation. For example, capacitances and resistances are very sensitive to changes in V_gs_ and V_ds_. Analysis of the intrinsic parameters shows that response times were faster and transconductance was better at lower negative gate voltages. These parameters are crucial for applications that require rapid switching. In the end, the equivalent-circuit model and the experimental data agreed very well with each other, indicating that the model is accurate in predicting the performance of a device. This study not only enhances our understanding of how GaN HEMTs function, but also provides valuable insights for developing more effective designs in cutting-edge RF integrated circuits.

## Figures and Tables

**Table 1:** Extrinsic parameter values of the GaN HEMT.

*V*_gs_ = 0 V and *V*_ds_ = 0 V (OFF Condition)	
Parameters	GaN HMET
L_g_ (pH)	142.0
L_s_ (pH)	1.80
L_d_ (pH)	64.0
R_g_ (Ω)	2.69
R_s_ (Ω)	3.10
R_d_ (Ω)	5.81
***V*_gs_ = −10.0 V and *V*_ds_ = 0 V (PINCH-OFF Condition)**	
C_pg_ (fF)	51.0
C_pd_ (fF)	85.0

**Table 2:** Quantitative comparison between simulated and experimental data (Figure 11).

Bias Condition (V_ds_, V_gs_)	RMSE (dB)	R^2^ Value
V_ds_ = 15 V, V_gs_ = −4.8 V	0.52	0.993
V_ds_ = 13 V, V_gs_ = −4.6 V	0.47	0.991
V_ds_ = 11 V, V_gs_ = −4.4 V	0.43	0.994
V_ds_ = 9 V, V_gs_ = −4.2 V	0.39	0.995

**Table 3:** DC, RF, and intrinsic parameters for various drain biases at V_gs_ = −4.8 V.

V_ds_ (V)	I_ds_ (mA)	g_m_ (mS)	C_gs_ (fF)	C_gd_ (fF)	C_ds_ (fF)	R_gs_ (Ω)	R_gd_ (Ω)	R_ds_ (Ω)	g_mo_ (mS)	τ_gm_ (ps)	τ_gs_ (ps)	τ_gd_ (ps)	f_t_ (GHz)	f_max_ (GHz)	S_21_ (dB)	Y_21_ (S)	K
3	21.81	45.45	192.51	55.62	163.79	1.08	13.04	166.01	67.96	2.27	1.31	4.55	45.81	84.72	12.70	0.0327	1.04
5	29.54	50.02	195.91	52.68	162.17	1.15	14.83	158.10	72.57	2.15	1.42	4.89	50.92	92.34	13.45	0.03093	0.97
7	37.73	61.25	197.27	49.16	160.65	1.23	16.63	177.12	73.77	2.17	1.52	5.13	51.62	96.01	13.63	0.03052	0.96
9	43.65	56.36	201.39	45.20	159.13	1.39	20.07	196.14	73.66	2.20	1.76	5.70	50.46	98.12	13.74	0.03049	0.96
11	47.75	56.88	205.21	42.21	157.56	1.46	21.87	201.70	72.90	2.24	1.89	5.79	48.80	98.90	13.75	0.03037	0.97
13	51.40	56.58	209.26	39.76	156.01	1.58	24.49	213.99	71.70	2.30	2.08	6.11	46.82	98.76	13.72	0.03041	0.99
15	54.71	55.83	212.77	37.65	154.46	1.70	27.11	226.28	70.26	2.35	2.27	6.41	44.83	98.16	13.66	0.03048	1.01

## Data Availability

The data presented in this study are available on request from the authors.
